# Translational Landscape of Nanopharmaceuticals: Progress and Prospects on Biomacromolecule Delivery and Active Targeting

**DOI:** 10.34172/apb.47355

**Published:** 2026-06-15

**Authors:** Azam Safary, Mostafa Akbarzadeh-Khiavi

**Affiliations:** ^1^Connective Tissue Diseases Research Center, Tabriz University of Medical Sciences, Tabriz, Iran; ^2^Research Center for Pharmaceutical Nanotechnology, Biomedicine Institute, Tabriz University of Medical Sciences, Tabriz, Iran; ^3^Liver and Gastrointestinal Diseases Research Center, Tabriz University of Medical Sciences, Tabriz, Iran

**Keywords:** Nanopharmaceuticals, Biomacromolecule delivery, Protein corona, Active targeting, Manufacturing scalability, Clinical translation

## Abstract

Over the past two decades, nanopharmaceuticals have demonstrated strong potential for biomacromolecule delivery, yet clinical translation remains scarce. This study examines the persistent gap between laboratory research and clinical application, drawing on practical lessons from enzyme-conjugated nanoparticles, EGFR-targeted gold nanoparticles, and magnetic nanobiosystems. Although multifunctional nanocarriers excel in vitro, their clinical success is often hindered by biological factors that are frequently neglected in laboratory studies. These issues include protein corona formation, which diminishes targeting specificity; complexities in optimizing ligand density; challenges in scaling up manufacturing; and regulatory hurdles. The protein corona, forming immediately after biological exposure, fundamentally alters nanoparticle identity and targeting in clinical environments. Moreover, complex multi-component designs often fail to translate due to batch variability and excessive production costs. This study advocates prioritizing design principles that emphasize manufacturability, biological robustness, and regulatory practicality over technological novelty. By applying insights from two decades of translational barriers, the field can advance toward clinically viable nanopharmaceutical platforms through simplified designs that address real-world limitations.

## Introduction

 Over the past two decades, the field of nanopharmaceuticals has experienced significant technological progress. Early predictions indicated that multifunctional nanocarriers would overcome biological barriers, improve pharmacokinetic profiles, protect against degradation, and enhance targeted cellular uptake.^1-3^ However, clinical outcomes have revealed that genuine therapeutic advancement requires more than laboratory-based technological superiority. It also requires a comprehensive understanding of biological constraints, manufacturing challenges, and practical design principles.

 A substantial gap persists between preclinical promise and clinical performance. Although academic laboratories continue to develop increasingly sophisticated nanoplatforms with notable in vitro efficacy, the rate of translation into approved clinical products remains low.^4,5^ This disconnect arises not from a lack of scientific innovation but from fundamental misalignments between research priorities and clinical needs. Our direct experience with enzyme-conjugated gold nanoparticles for combination therapy,^6^ EGFR-targeted systems for KRAS-mutant colorectal cancer,^7^ and immobilized enzyme platforms on magnetic nanoparticles has provided critical insights into the factors that determine clinical success.^8^

 This perspective, informed by hands-on experience with three distinct nanoplatform families, examines the challenges inherent in the bench-to-bedside transition. Instead of cataloging technological achievements, we emphasize the practical barriers encountered during translation and the design principles that emerge from addressing them. Our objective is not to undervalue fundamental research, but to advocate for development strategies aligned with clinical requirements from the earliest stages of design.

## Challenges in Biomacromolecule Delivery

 Therapeutic proteins, such as enzymes, antibodies, and nucleic acids, represent a significant and expanding segment of contemporary therapeutics.^9,10^ These biomacromolecules exhibit high specificity and potent biological activity. However, they face substantial delivery challenges. These challenges include rapid enzymatic degradation in biological fluids, premature renal clearance due to small molecular size, limited ability to traverse biological barriers, and restricted tissue distribution.^11,12^ Even highly potent therapeutic agents are rendered clinically ineffective if they are degraded or eliminated before reaching their target sites.

 Nanocarrier systems have been proposed as solutions to these fundamental challenges.^13^ Encapsulation or conjugation of therapeutic biomacromolecules to nanoparticles can provide protection against degradation. These strategies can also extend circulation time and enhance tissue accumulation.^14,15^ Studies with enzyme-based nanocarrier systems indicate that preserving biological activity during surface attachment and throughout biological distribution, while maintaining protective benefits, requires careful optimization.

 Research on gold nanoparticle systems conjugated with therapeutic enzymes has demonstrated that conjugation chemistry significantly influences enzymatic function.^16^ Covalent attachment strategies should preserve active site accessibility and maintain protein conformational stability. Investigations using PEGylated silica-coated magnetic nanoparticles for enzyme immobilization have shown that optimizing enzyme stability and catalytic accessibility requires systematic adjustment of surface chemistry, spacer length, and attachment density.^17^ Enzyme activity may decrease by 40-60% following conjugation if attachment chemistry is not carefully optimized. However, this loss can be minimized through rational linker design and controlled conjugation conditions. These practical considerations are often underestimated in proof-of-concept studies but become critical during scale-up and clinical translation.

## The Protein Corona Reality

 Upon exposure to biological fluids, nanoparticles rapidly adsorb serum proteins, resulting in the formation of a protein corona.^18,19^ This dynamic layer, composed of hundreds of proteins with varying affinities and exchange rates, determines the biological identity of the nanoparticle.^20^ Despite the application of targeting moieties and stealth polymers, the protein corona consistently modifies surface properties, particle size, charge, and, notably, the accessibility of targeting ligands in most nanoparticle designs.^21,22^

 Studies on protein corona dynamics have shown that corona composition depends on nanoparticle surface chemistry, size, charge, shape, biological fluid composition, and exposure duration.^23,24^ The protein corona exists in a dynamic equilibrium, with proteins continuously adsorbing and desorbing. Initially, low-affinity ‘soft corona’ proteins are gradually replaced by ‘hard corona’ proteins with higher affinity for the nanoparticle surface.^25^

 This phenomenon significantly impacts nanoparticle targeting. Even precisely engineered targeting ligands may become partially or completely obscured by adsorbed proteins, diminishing their clinical effectiveness.^26^ Although pre-coating nanoparticles or modifying their surfaces can influence corona formation, complete prevention remains unattainable.^27^ Additional studies report that corona formation can reduce ligand accessibility by 50-70%, depending on nanoparticle characteristics and biological conditions.^28^ Therefore, effective targeting strategies must consider the unavoidable presence of protein coronas rather than assuming nanoparticles maintain pristine surfaces at target tissues.

## Active Targeting: Promise and Reality

 Active targeting, which involves decorating nanoparticles with ligands that recognize specific cellular receptors, has been a central strategy in nanomedicine.^29,30^ The theoretical benefit is the selective accumulation of nanoparticles at disease sites, potentially enhancing efficacy and reducing systemic toxicity. However, experience with EGFR-targeted gold nanoparticles for KRAS-mutant colorectal cancer has revealed fundamental challenges in translating this concept to clinical practice.^31^

 Although EGFR overexpression in cancer cells offers a theoretical targeting advantage, optimizing ligand density remains challenging. Insufficient ligand density reduces targeting efficiency and cellular uptake, while excessive density leads to rapid immune clearance, increased non-specific binding to healthy tissues, and potential receptor-mediated toxicity.^29^ The optimal density range is narrow and depends on nanoparticle size, surface chemistry, ligand type, and target cell receptor expression. Supporting these observations, Wilhelm et al. reported that only 0.7% (median) of the administered nanoparticle dose reaches solid tumors, underscoring the limitations of current targeting strategies.^32^ Furthermore, our EGFR-targeted systems demonstrated that receptor-mediated endocytosis, though successful in controlled in vitro experiments, faces substantial competition from protein corona proteins in vivo, reducing targeting specificity.^33^ The enhanced permeability and retention (EPR) effect, long considered foundational for nanoparticle tumor accumulation, shows substantial inter-patient variability and appears to be less reliable in human tumors than in rodent models in most clinical settings.^34,35^ Recent clinical analyses and reviews support this observation, suggesting that EPR-based strategies alone are insufficient for reliable therapeutic efficacy.^36-38^

## Multifunctional Designs: Complexity as a Barrier

 The pursuit of multifunctional nanocarriers—materials designed to transport drugs in the body that combine precise drug delivery to targeted tissues (targeting), allow medical imaging (imaging capabilities), respond to specific stimuli like pH or temperature (stimulus responsiveness), and carry several therapeutic drugs at once—reflects scientific ambition but often hinders clinical translation.^39^ Research on these systems, which combine treatment and diagnostic functions, has shown that each added feature introduces greater variability in their design, increases the difficulty of thoroughly analyzing them, and creates uncertainty in the approval process.^40,41^

 From a practical perspective, complex designs face exponentially increasing quality control requirements.^42^ Each unique function added to the nanocarrier—which includes features such as targeting or imaging—needs its own testing to confirm it works reliably, and all possible interactions must be checked under different conditions. Ensuring that each batch of nanoparticles is the same becomes harder as more functions are added.^43^ Manufacturing guidelines must guarantee not just that each feature works, but also that its proportions and arrangement within each tiny particle remain consistent from one batch to another.^40^

 Regulatory agencies demand comprehensive characterization of each functional element and its interactions, which substantially extends approval timelines and increases development costs.^44,45^ Clinical trials must show that each component provides meaningful therapeutic benefit, not just technological sophistication.^46^ Historical analysis of approved nanomedicines reveals that simplified designs with clearly defined mechanisms and reproducible manufacturing processes succeed more often than complex platforms requiring extensive optimization.^47,48^

## Manufacturing and Scalability Challenges

 Laboratory-scale synthesis methods—techniques used to produce small amounts in research labs—often do not perform as well when adapted for industrial-scale production.^49^ Methods that yield uniform, well-defined nanoparticles in small (milligram) quantities often produce mixed-size particles with variable properties when scaled up to the much larger (kilogram) amounts needed for clinical testing.^43^ Our research on making magnetic nanoparticles for attaching enzymes (enzyme immobilization) showed that keeping the size distribution, surface chemical makeup, and density of attachment points the same at larger scales needs a lot of adjustment and improvement in the process.^50^

 Synthesis methods often used in academic research—such as those involving organic (carbon-based) solvents, poisonous chemicals, or complex multi-step cleaning processes—become extremely expensive or impractical for commercial or clinical production.^51^ Producing materials in sterile conditions, as required by regulations for human use (known as Good Manufacturing Practice, or GMP), further increases costs and practical difficulties.^52^ Every ingredient used must be approved (qualified), every step in the process must be confirmed to work as intended (validated), and every test method needs thorough checking (verification).^44^

 The economics of manufacturing cannot be ignored.^53^ Production costs must align with reimbursement realities and competitive pricing pressures. A nanoformulation that requires exotic materials, multi-step synthesis, extensive purification, and complex quality control faces substantial economic barriers, regardless of therapeutic efficacy.^48^ Formulation stability during storage, transportation, and clinical administration presents additional hurdles often underestimated in academic research focused on freshly prepared formulations.^54^

## Regulatory and Economic Realities

 Regulatory approval pathways for complex nanoformulations remain challenging and evolving.^42,55^ Each nanoparticle component needs separate toxicological evaluation, pharmacokinetic assessment, and stability testing. Combination products face extra regulatory scrutiny. For approval, it must be shown that each component provides a meaningful therapeutic benefit rather than merely increasing complexity.^56^

 Characterization requirements for nanopharmaceuticals exceed those for conventional small-molecule drugs.^44^ Physicochemical properties, including size distribution, surface charge, morphology, and chemical composition, must be rigorously characterized. Biological properties, including protein binding, cellular uptake mechanisms, biodistribution, and immunogenicity, require extensive evaluation.^45^ Any changes in manufacturing processes necessitate comparability studies demonstrating that product properties remain consistent with FDA and EMA guidelines.^56,57^

 Economic viability demands consideration beyond scientific innovation.^53^ Development costs for nanopharmaceuticals are substantially higher than those for conventional small-molecule drugs. Manufacturing complexity, extensive characterization requirements, specialized quality control, and prolonged regulatory timelines create financial barriers that scientific merit alone cannot overcome.^48^ Market size, reimbursement policies, and competitive landscape must support the substantial investment required for clinical development.^53^

## Lessons for Future Development

 Over the past two decades, nanomedicine research has generated insights that will advance future research. Simplicity in nanomedicine design should be prioritized. Reducing the number of components streamlines manufacturing. It also accelerates regulatory approval and lowers costs.^58^ Each component should be justified by a clear benefit. Unnecessary elements must be eliminated without compromising efficacy. Development decisions should rely on streamlined strategies.^59^ Once a simple and effective design is in place, manufacturability becomes the next critical step.

 After establishing a streamlined design, focus on manufacturability from the outset. Synthetic methods must suit industrial scale-up. Raw materials should be commercially available, cost-effective, and consistent.^60^ Scalable analytical methods compatible with automation must ensure reliable quality control.^42^ Early attention to these aspects will facilitate subsequent development. After optimizing manufacturing processes, address biological challenges.^44^

 After defining manufacturing approaches, tailor design strategies to address biological realities. Evaluate protein corona formation, immune recognition, and biological barriers in vivo instead of relying solely on in vitro studies.^26^ Targeting strategies must show clear advantages over passive accumulation in relevant animal models. Use clinically relevant doses and administration routes in pharmacokinetic and biodistribution studies.^36^ Once biological challenges are addressed, identify the clinical contexts for greatest impact.

 After resolving biological challenges, focus on three areas. First, address unmet clinical needs, especially where nanocarriers offer major advantages over existing therapies. Second, target diseases that lack effective treatments or those currently managed by drugs with narrow therapeutic windows.^4,46^ Third, clarify structure-activity relationships, identify critical quality attributes, and establish robust design spaces to enable ongoing improvement.^47,61^ Table 1 summarizes the key translational barriers encountered across our experimental systems along with the proposed pragmatic design strategies.

**Table 1:** Key Translational Barriers Encountered in Nanopharmaceutical Systems and Proposed Design Strategies

**Translational Challenge**	**Observation in Our Systems**	**Clinical Consequence**	**Pragmatic Design Strategy**
Protein Corona Formation	Immediate adsorption of serum proteins altered targeting specificity of EGFR-targeted gold NPs in physiological conditions	Loss of active targeting efficiency; altered biodistribution and immune recognition	Design corona-resistant surface coatings (e.g., zwitterionic polymers); incorporate protein corona profiling in early preclinical stages
Ligand Density Optimization	Suboptimal anti-EGFR antibody density on silica-coated magnetic NPs reduced binding affinity in KRAS-mutant CRC cells	Insufficient receptor engagement; decreased therapeutic efficacy	Systematic ligand density screening under biologically relevant conditions prior to scale-up
Manufacturing Scalability	Batch-to-batch variability in enzyme-conjugated gold NP synthesis compromised reproducibility	Inconsistent product quality; regulatory non-compliance risk	Prioritize simple, reproducible conjugation chemistries; integrate scale-up feasibility from initial design phase
Biological Stability	Enzyme activity loss upon nanoparticle conjugation under physiological pH and temperature	Reduced catalytic and therapeutic performance in vivo	Optimize immobilization conditions; validate enzymatic activity retention across storage and physiological conditions
Regulatory Complexity	Multi-component nanoplatform characterization requirements exceed standard pharmaceutical frameworks	Prolonged approval timelines; high development costs	Minimize formulation complexity; align with existing regulatory guidance (FDA/EMA nanomedicine frameworks)
Economic Viability	High production costs of multifunctional nanocarriers limit industrial scalability	Limited commercial interest; restricted patient access	Design cost-effective platforms with fewer components without compromising therapeutic performance

Observations are derived from direct experimental experience with enzyme-conjugated nanoparticles [6,8], EGFR-targeted gold nanoparticle systems [7], and magnetic nanobiosystems [7,8], as discussed in the text.

## Conclusion

 The nanopharmaceutical field stands at a critical juncture. Academic researchers continue to develop sophisticated platforms with impressive in vitro results. However, clinical translation remains limited.^36,62^ This disconnect reflects not a lack of scientific ingenuity, but a misalignment between research priorities and clinical requirements.^63^ To make progress, honestly assess past challenges and commit to pragmatic design principles.

 Experience with enzyme-conjugated nanoparticles, EGFR-targeted gold nanoparticle systems, and magnetic nanobiosystems shows that clinical success requires more than technological sophistication. It also demands manufacturing feasibility, biological stability in complex physiological environments, regulatory compliance, and economic viability.^42,53^ Designers cannot ignore the protein corona; it must be accommodated in strategies.^26^ Manufacturing scale-up challenges cannot be postponed to later stages. They must inform initial design choices.^43^

 The objective should be to develop clinically viable solutions that address genuine therapeutic needs. Avoid pursuing technological complexity for its own sake.^47^ Collaboration among academic researchers, industrial partners, regulatory agencies, and clinicians should start early.^63^ Assess biological barriers regularly in real-world contexts, not only in laboratory settings.

 By using lessons from two decades of translational challenges and adopting simple, manufacturing-conscious designs, nanomedicine can fulfill its therapeutic promise.^4^ Instead of adding functional components or seeking ever more complex platforms, focus on robust, reproducible systems that transition from the laboratory to the patient’s bedside. ^46^ Success should come from pragmatic designs that prioritize clinical viability over technological virtuosity. Deliver real therapeutic benefits to patients who need them most. Future funding and publication priorities should explicitly reward translational feasibility rather than technological complexity.^36^

## Availability of Data and Materials

 Not applicable.

## Competing Interests

 The authors declare that they have no known competing financial interests or personal relationships that could have appeared to influence the work reported in this paper.

## Consent for Publication

 All authors have approved to publish this manuscript.

## Data Availability

 Not applicable.

## Ethical Approval

 Not applicable.
